# Lung volume reproducibility under ABC control and self‐sustained breath‐holding

**DOI:** 10.1002/acm2.12034

**Published:** 2017-02-25

**Authors:** Evangelia Kaza, Alex Dunlop, Rafal Panek, David J. Collins, Matthew Orton, Richard Symonds‐Tayler, Dualta McQuaid, Erica Scurr, Vibeke Hansen, Martin O. Leach

**Affiliations:** ^1^ CR‐UK Cancer Imaging Centre Institute of Cancer Research and Royal Marsden Hospital London UK; ^2^ Joint Department of Physics The Institute of Cancer Research and Royal Marsden Hospital London UK; ^3^ The Royal Marsden NHS Foundation Trust London UK

**Keywords:** ABC, breath‐hold, lung volume reproducibility

## Abstract

An Active Breathing Coordinator (ABC) can be employed to induce breath‐holds during CT imaging and radiotherapy of lung, breast and liver cancer, and recently during lung cancer MRI. The apparatus measures and controls respiratory volume, hence subject lung volume reproducibility is its principal measure of effectiveness. To assess ABC control quality, the intra‐session reproducibility of ABC‐induced lung volumes was evaluated and compared with that reached by applying the clinical standard of operator‐guided self‐sustained breath‐holds on healthy volunteers during MRI. Inter‐session reproducibility was investigated by repeating ABC‐controlled breath‐holds on a second visit. Additionally, lung volume agreement with ABC devices used with different imaging modalities in the same institution (MR, CT), or for a breast trial treatment, was assessed. Lung volumes were derived from three‐dimensional (3D) T1‐weighted MRI datasets by three observers employing semiautomatic lung delineation on a radiotherapy treatment planning system. Inter‐observer variability was less than 6% of the delineated lung volumes. Lung volume agreement between the different conditions over all subjects was investigated using descriptive statistics. The ABC equipment dedicated for MR application exhibited good intra‐session and inter‐session lung volume reproducibility (1.8% and 3% lung volume variability on average, respectively). MR‐assessed lung volumes were similar using different ABC equipment dedicated to MR, CT, or breast radiotherapy. Overall, lung volumes controlled by the same or different ABC devices agreed better than with self‐controlled breath‐holds, as suggested by the average ABC variation of 1.8% of the measured lung volumes (99 mL), compared to the 4.1% (226 mL) variability observed on average with self‐sustained breath‐holding.

## 1 Introduction

Imaging and radiotherapy of the thorax and abdomen are adversely affected by respiratory motion. Breath‐holding for short time intervals is a widely applied technique to reduce this effect. In standard clinical practice, patients are instructed to hold their breath,^1^ yet the true onset, constancy and reproducibility of individual self‐sustained respiration control is questionable, especially in the absence of respiratory monitoring, and depends on patient compliance. To address this issue, a volumetric respiratory monitoring and control apparatus that induces breath‐holds automatically at a predefined inhaled or exhaled air volume during a preset duration (active breathing coordinator, ABC) has been developed by Elekta (Elekta Oncology Systems Ltd, Crawley, West Sussex, UK). The ABC can be employed during lung,^2^ breast^3^ and liver radiotherapy^4^ to minimize breathing motion and consequently to reduce treatment margins and/or to reduce dose to healthy tissue. An ABC consists of a breathing tube with a mouthpiece and filter, connected to a digital volume transducer & pickup assembly and a balloon valve that can be inflated to halt respiration.^5^ The goal of the device is to induce reproducible breath‐holds at the same lung volume, during and across sessions, whether this may be radiotherapy delivery or CT or PET examinations. Lung volume reproducibility is therefore the main characteristic of the ABC respiratory control effectiveness.

In addition to CT, PET, and radiotherapy, ABC has been recently used in an MR setting.^6^ ABC employed in abdominal MRI of healthy volunteers indicated better organ position reproducibility and improved image quality compared to repeated self‐induced breath‐holding. Measuring the lung volume reproducibility achieved with these breath‐holding methods would characterize the ABC advantages. Kaza et al.^6^ also demonstrated that lung cancer MR images acquired under ABC control with identical ABC settings and patient positioning as employed during radiotherapy registered well with ABC‐controlled CT images acquired 2 weeks earlier. The quality of MR‐CT inter‐modality agreement is crucial for extracting additional information to CT from the various contrast mechanisms achieved with MRI (T_1_, T_2_, diffusion weighting); examples include detection and characterization of pulmonary nodules, differentiation of lung cancer from secondary changes and assessment of mediastinal invasion and lymph node involvement.^7^ Moreover, changes in apparent diffusion coefficient (ADC) values calculated from diffusion‐weighted MRI (DW‐MRI) at different treatment stages may indicate tumor response.^8^ As ABC control offers the potential to produce well‐matched images during different acquisition sessions, it is important to assess lung volume consistency between such sessions. In clinical practice, similar ABC devices of the same model are dedicated to different imaging or treatment modalities, thus air volume repeatability should also be compared between these devices.

In an ABC feasibility study, McNair et al.^9^ have observed that outlined lung volumes on planning CT scans agreed with tidal volumes recorded by the ABC, but that the ABC‐displayed breath‐held volumes were affected by flow rate. As this effect may raise concerns about the reproducibility of the air volumes controlled by the ABC, actual lung volume measurements on human subjects are necessary for clarification. Treating breast cancer patients, Bartlett et al.^10^ found no significant differences in lung volumes between ABC control and a specific voluntary breath‐holding technique where breath‐hold consistency was monitored by the position of patient tattoos relative to lasers. Using a different spirometer, Fassi et al.^11^ found that spirometer‐based control did not guarantee a reproducible position of the external breast surface in deep inhalation breath‐hold (DIBH) left‐breast radiotherapy. Nevertheless, measuring whole lung volumes from three‐dimensional (3D) imaging would present a more accurate evaluation of spirometer‐based respiration control than external markers. Hunjan et al.^12^ have also observed that abdominal external fiducial extrema positions differed between breath‐holding and free breathing.

Single organs of interest have primarily been assessed during radiotherapy: Eccles et al.^4^ have observed a good intra but less satisfactory inter‐fraction reproducibility of liver position using ABC; employing a different active breathing control device for breast radiation therapy, Moran et al.^13^ have shown that short‐term reproducibility of nodal target position was ≤0.4 cm for breath‐holds in different respiratory phases. However, assessing the reproducibility of the entire lung volume provides a generic measure of respiration control, valid for various regions inside or around the lungs. Regarding voluntary breath‐holding, Starkshall et al.^14^ observed a similar inter‐fractional lung tumor location reproducibility compared to gating. Hanley et al.^15^ have shown a 2.5 times higher inter breath‐hold than intra‐breath‐hold variation by measuring the reproducibility of diaphragm position during verbally coached voluntary DIBH maneuvers.

The present work serves as a quality assurance of clinically used ABC devices during clinically applicable imaging of human subjects without radiation exposure. We evaluated intra‐session and inter‐session lung volume reproducibility achieved with ABC control, by repeating breath‐holds with the modified MR‐compatible ABC system applied to lung cancer imaging.^6^ The study was performed on healthy volunteers, assuming no significant physiological changes in the course of one to 4 weeks between two imaging sessions. Moreover, we investigated if similar ABC devices used with different clinical imaging modalities (MR, CT, breast trial radiotherapy) reliably provide the same lung volumes. In addition, we examined whether ABC provides more reproducible lung volumes than the clinical standard of self‐sustained breath‐holding, where an operator instructed each volunteer to perform repeated self‐controlled breath‐holds. In all cases, lung volume measurements were extracted from 3D T_1_‐weighted volumetric MR images acquired during each breath‐hold.

## 2 Methods

### 2.A ABC set up

We used the modified MR‐compatible ABC system described by Kaza et al.,^6^ consisting of the ABC respiratory system (breathing tube with filter, digital volume transducer & pickup assembly, balloon valve, and couplers), modified MR alert bulb and adapter as well as custom connectors and balloon valve tube extension. The digital volume transducer & pickup assembly, balloon valve, and 2.25 m pneumatic tubing dedicated for MR application were denoted as “MR‐ABC kit.” The corresponding components of the ABC apparatus used in our institution during CT lung treatment planning scans and of the ABC device used for treating breast cancer patients participating in a trial were borrowed for comparison and named “CT‐ABC kit” and “trial‐ABC kit,” respectively.

A modified Extended Wing Board used for radiotherapy (Oncology Systems Limited, Shropshire, UK) was employed for volunteer positioning. The ABC breathing tube with filter on one side of a custom post was connected to the easily interchangeable MR‐ABC, CT‐ABC or trial‐ABC kit on the other side of the post. A 3.05 m balloon valve pneumatic tube extension and a custom electrical cable was attached to each kit, feeding into the ABC control module through the radiofrequency (RF) penetration panel. The module was connected via a serial cable to a computer executing the ABC 2.00 control software in the scanner control room. The set up was similar to,^6^ except that no external triggering circuits were used in the present work.

### 2.B Volunteer measurements

Six healthy volunteers (two females, four males, age range 25–58 yrs, height range 1.65–1.93 m) were trained for ABC use before MR scanning. The study was approved by the Institutional Review Board and all subjects provided their informed consent. Volunteers were familiarized with the device and asked to breathe in to their maximum capacity three times. The mean of the maximum volume reached during each of these deep inhalations was considered as the deep inspiration volume (DIV) of the volunteer. A threshold of 75% DIV was set in the ABC control software to induce breath‐holds on moderate deep inspiration, as is customary during lung radiotherapy^2^ and breast radiotherapy.^3^ To ensure participant safety, volunteers practiced an ABC‐controlled breath‐hold of the intended duration at their individual 75% DIV level, and aborting such a breath‐hold by pressing the modified MR alert bulb. Subsequently, subjects lay on the Extended Wing Board placed on the scanner couch. The individual optimal handle bar position, and padding to comfortably support their arms superior to their head, was determined and noted for use in later measurements. A body matrix array coil was placed on the volunteers’ chest; a total of 6 anterior and 6 posterior coils were employed. When positioned in the scanner they were breathing through the ABC respiratory system with their nose clamped. The subjects received no visual feedback of their respiratory traces.

An operator monitored the subjects’ respiratory traces on the ABC control laptop. To perform a volumetric MR acquisition in an ABC‐controlled breath‐hold, the operator first prepared the measurement on the scanner console configured to require a user prompt to start acquisition. The operator then activated the ABC and instructed the volunteer to take a deep breath in when ready. When the inhaled volume exceeded the individually defined 75% DIV threshold and reached a plateau due to the balloon valve closing, the operator commenced MR scanning. The remaining breath‐holding time was counted down to the volunteer. A 3D T_1_‐weighted volumetric interpolated breath‐hold examination (VIBE) sequence (TR 4 ms, TE 0.93 ms, FoV 299*399 mm^2^, acquisition matrix 324*576 interpolated, flip angle 8°, acceleration factor GRAPPA3) was acquired axially during the single breath‐hold. Whole lung coverage was achieved with 112 partitions of 3 mm thickness, except for tallest subject, Volunteer 4, who required 120 partitions. The actual breath‐hold duration equaled the acquisition time of 22.5 or 25 s, respectively.

The VIBE measurement was repeated four times in total with the MR‐ABC kit for each volunteer, to assess intra‐session lung volume reproducibility with this kit. The measurement was repeated four times with the CT‐ABC and trial‐ABC, in order to assess intra‐session lung volume reproducibility with each of these kits and to investigate possible kit‐related variations. The ABC kits were exchanged by an operator as described in section A, without moving the volunteer. For comparison with self‐induced suspension of respiration, the VIBE measurement was also acquired four times in self‐controlled breath‐holds using the ABC spirometer. The scanner was then prepared as before and subjects were instructed to “breathe in, breathe out, breathe in, breathe out, breathe in and hold” as in common clinical practice, whilst an operator observed their respiratory volume traces. Volunteers were given no instructions regarding the inhalational level of self‐sustained breath‐holds. Similarly to the ABC‐controlled case, the operator prompted MR image acquisition when the inhaled volume reached its breath‐holding plateau. Upon completion of a breath‐hold, volunteers were instructed to breathe normally.

For inter‐session lung volume reproducibility assessments, the VIBE measurement was acquired four times for each volunteer on a second session, using the MR‐ABC kit and the same individual volume threshold, arm padding and positional settings in order to preserve volunteer position as far as possible. The interval between the two sessions varied from 1 week to 1 month. In total, twenty 3D image datasets were produced for every volunteer, arising from the four repeat acquisitions performed for each of the five conditions: MR‐ABC on session 1, MR‐ABC on session 2, CT‐ABC, trial‐ABC, and self‐controlled breath‐holding.

### 2.C Lung volume calculations

A semi‐automated lung contouring method was applied using the RayStation (RayStation v4.6, RaySearch Laboratories AB, Stockholm, Sweden) radiotherapy treatment planning system (TPS). Lung regions of interest (ROIs) were initially segmented using a thresholding technique applied to the 3D T_1_‐weighted image datasets and cleaned by removing holes and small contours (<0.1 cm^3^). The automatically generated ROIs for each 3D dataset were then manually edited to account for disagreements between the threshold‐generated volume and visually perceived lung borders. Manual editing was performed on each axial partition whilst observing, and taking into account, all three orthogonal orientations. The contours of a segmented lung volume were subsequently smoothed by applying a 5 mm isotropic expansion followed by contraction by the same amount. Finally, the smoothed contours were visually examined and manually edited when necessary. The resulting volume for each T_1_‐weighted image dataset was considered as the volume of the entire lungs for the corresponding breath‐hold and used for further analysis.

For efficient evaluation the 120 acquired image datasets were equally divided between three physicists (Observer 1–3). Observer 1 delineated the entire lung volumes on all 3D image datasets of Volunteers 1 and 2, while Observers 2 and 3 processed the data of Volunteers 3 and 4, and 5 and 6, respectively. In order to evaluate inter‐observer variability, all three observers additionally independently contoured an image data batch comprising the four 3D acquisitions of a specific breath‐holding condition from two different subjects, whilst blinded to the results of the other observers. Batch A (Volunteer 6, MR‐ABC session 2) had an image quality representative of most MR measurements of this study. Batch B (Volunteer 4, MR‐ABC session 2) presented the worst case, featuring a ghosting artifact that increased the amount of manual corrections required to precisely delineate the lung ROI. To estimate the delineation variability between observers, the percentage lung volume difference between each pair of observers for each of the four 3D image datasets of these two batches was calculated. The mean and standard deviation of these differences over the four image datasets of each batch represented the bias and variation, respectively, between observers for this data.

### 2.D Statistical volume analysis

Lung volume reproducibility assessments were performed on the data calculated from the delineated lung ROIs on the 3D MR image datasets. The mean and standard deviation of the four breath‐holding volume values of each condition were computed for every subject. To compare the ABC‐controlled respiratory levels between conditions per subject, we calculated the percentage difference of the mean lung volume of an ABC breath‐holding condition from the mean lung volume of MR‐ABC session 1, selected as reference. Kit intra‐session lung volume variation was defined as the mean of the standard deviations of the delineated volumes over all subjects for each ABC kit separately. Overall ABC intra‐session lung volume variation was defined as the mean of the standard deviations over all subjects and ABC conditions. Self‐sustained intra‐session lung volume variation was represented by the mean of the standard deviations of the delineated volumes over all subjects for self‐controlled breath‐holding. Inter‐session lung volume variation was defined as the mean absolute lung volume difference over all volunteers measured with the MR‐ABC kit on different dates.

In order to investigate lung volume agreement between the different ABC breath‐holding conditions over all subjects, Bland‐Altman analysis^16^ was performed. The means of the four breath‐holding lung volumes for each ABC condition and volunteer were used as input data, to improve the accuracy of the method. We compared the mean volumes between the two MR‐ABC sessions over volunteers, and the average volume of both sessions to the other ABC conditions. The mean lung volumes of four breath‐holds achieved with the CT‐ABC were also compared to those achieved with the trial‐ABC kit.

## 3 Results

### 3.A Lung volume delineation

**Figure 1:**
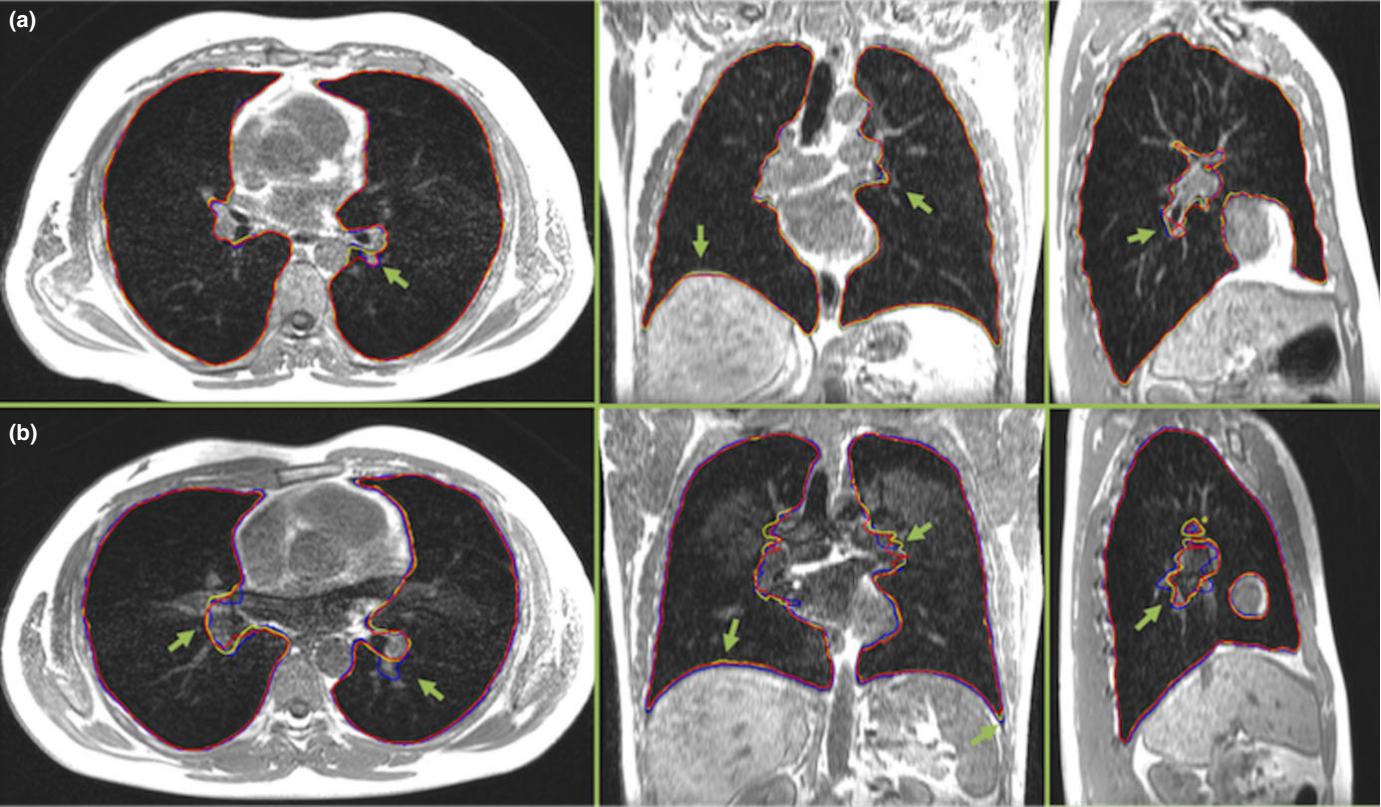
(a) An example partition from a 3D T_1_‐weighted image dataset (Volunteer 6, session 2), in the acquired axial and reconstructed coronal and sagittal orientation, during an MR‐ABC controlled breath‐hold. This image quality was typical for most acquisitions. The lung ROIs delineated by Observer 1, 2, and 3 appear in yellow, blue and red, respectively. (b) A similar example partition (Volunteer 4, session 2), presenting the worst image quality of this work due to a ghosting artifact. All images in (a) and (b) are equally windowed. The arrows point to instances of discrepancies between observers, which occurred mostly around the blood vessels and diaphragm. The original images were slightly cropped for display.

Figure 1 depicts the lung contours determined by all three observers on a partition of a breath‐holding 3D image dataset from one batch with typical and one with the worst image quality perceived in this investigation. The difference in calculated lung volumes for the 3D image dataset with typical image quality displayed in (a) amounted to 0.3%, 1.8% and 1.5% between Observers 1 & 2, 1 & 3, and 2 & 3, respectively. The equivalent lung volume differences for (b) with the worst image quality were 5.0%, 0.2%, and 4.5%.

The limits of agreement on lung volume calculations between the three observers, expressed as mean (bias) ± 1.96 standard deviations (reference interval) of the percentage of their volume differences, are shown in Table 1 for each of the two batches comprising four 3D image datasets. The mean absolute bias between observers 1 and 2 (0.5%) is smaller than the bias between observers 1 and 3 (2.7%) and observers 2 and 3 (3.0%). The average absolute bias and reference interval from all observers and image datasets was 2.1 ± 3.7%.

**Table 1:** Bias (mean) ± reference interval (1.96*standard deviation) of the percentage lung volume differences between observers

	% difference Observer 1 – 2	% difference Observer 1 – 3	% difference Observer 2 – 3
Batch A	0.2 ± 0.4	3.9 ± 4.4	3.7 ± 4.3
Batch B	−0.8 ± 5.5	1.5 ± 3.4	2.3 ± 4.5

batch A: four 3D T1‐weighted acquisitions in MR‐ABC breath‐holds with representative image quality; batch B: four 3D T1‐weighted acquisitions in MR‐ABC breath‐holds presenting the worst image quality of this study.

### 3.B Lung volume reproducibility of breath‐holding conditions

Table 2 demonstrates the mean and standard deviation of the delineated volumes from the four 3D image datasets with the same breath‐holding condition for each subject. Kit intra‐session variability of measured lung volumes was similar for the two MR‐ABC sessions (98 mL and 97 mL), and comparable to that for CT‐ABC and trial‐ABC (112 mL and 91 mL, respectively). The average MR‐ABC kit intra‐session variability from the two visits was 1.8% of the delineated lung volumes. The overall ABC intra‐session lung volume variation, as expressed by the average standard deviation of all ABC conditions over all volunteers, was 99 mL, representing 1.8% of the mean lung volume under ABC control. In contrast, the average standard deviation of the self‐sustained breath‐holds over subjects (self‐sustained intra‐session lung volume variation) amounted to 4.1% of the mean self‐controlled volume (226 mL). In general, volume standard deviation ranged from 33 to 185 mL under ABC control, but from 105 to 594 mL for self‐induced respiration holds. The absolute lung volume difference between the two MR‐ABC sessions was 3% on average, suggesting good inter‐session lung volume reproducibility. The CT‐ABC and trial‐ABC volumes differed by 2 and 5% on average from MR‐ABC session 1, also indicating good lung volume reproducibility between the three ABC kits.

**Table 2:** Mean ± standard deviation of delineated lung volumes (mL) for each volunteer and breath‐holding condition

*Volunteer*	*MR‐ABC 1*	*MR‐ABC 2*	*CT‐ABC*	*Trial‐ABC*	*Self‐BH*
1	4750 ± 70 *n.d*.	4843 ± 103 *−2%*	4632 ± 90 *2%*	4568 ± 90 *4%*	4894 ± 133 *n.d*.
2	4619 ± 100 *n.d*.	4504 ± 119 *3%*	4518 ± 150 *2%*	4293 ± 71 *7%*	4636 ± 105 *n.d*.
3	6038 ± 138 *n.d*.	6045 ± 33 *0%*	5973 ± 61 *1%*	5835 ± 76 *3%*	4336 ± 203 *n.d*.
4	5538 ± 119 *n.d*.	5760 ± 34 *−4%*	5412 ± 100 *2%*	5401 ± 48 *2%*	5524 ± 186 *n.d*.
5	6252 ± 77 *n.d*.	6181 ± 108 *1%*	6251 ± 114 *0%*	6082 ± 116 *3%*	6361 ± 594 *n.d*.
6	6065 ± 84 *n.d*.	5687 ± 185 *6%*	5674 ± 159 *6%*	5383 ± 143 *11%*	6796 ± 137 *n.d*.

Italics indicate the percent difference of the mean lung volume of each ABC specific breath‐holding condition from the mean lung volume of MR‐ABC session 1.

*n.d*.: not defined.

**Figure 2:**
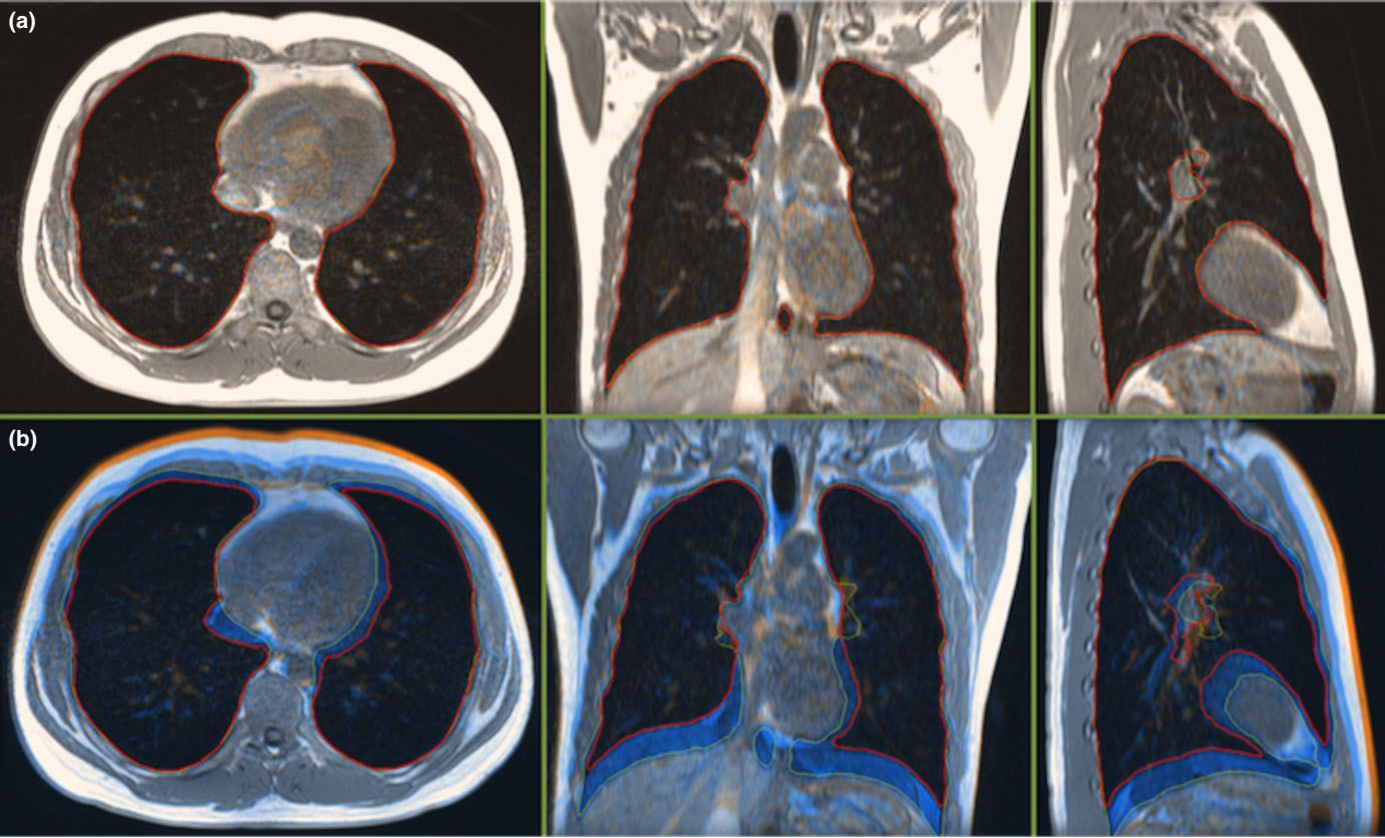
(a) A partition of the 3D image datasets of BH1 and BH4 of condition MR‐ABC 2 for Volunteer 5, with a small difference (4 mL) between their delineated lung volumes. Images are displayed in the acquired axial and reconstructed coronal and sagittal orientation. The image dataset of BH4 was rigidly registered on the BH1 dataset. The solid red and dotted yellow lines represent the delineated lung volume of BH1 and BH4, respectively. The images and lung contours demonstrate an excellent agreement. (b) A similarly positioned partition of self‐sustained BH4 rigidly registered on self‐sustained BH2 for the same volunteer, with a high difference (1370 mL) between the two delineated lung volumes, displayed in all three orientations. The images of the two breath‐holds present notable discrepancies in diaphragm, vessels and thorax position, displayed as blue and orange shaded regions.

Figure 2 illustrates an example of the spatial overlap between breath‐holding volumes with a (a) small or (b) high lung volume difference at a similar slice position in all three orientations for the same volunteer. For each of the two displayed pairs of image datasets with delineated lung volumes, the second 3D image dataset was rigidly registered on the first 3D image dataset and their differences were visually assessed.

**Figure 3:**
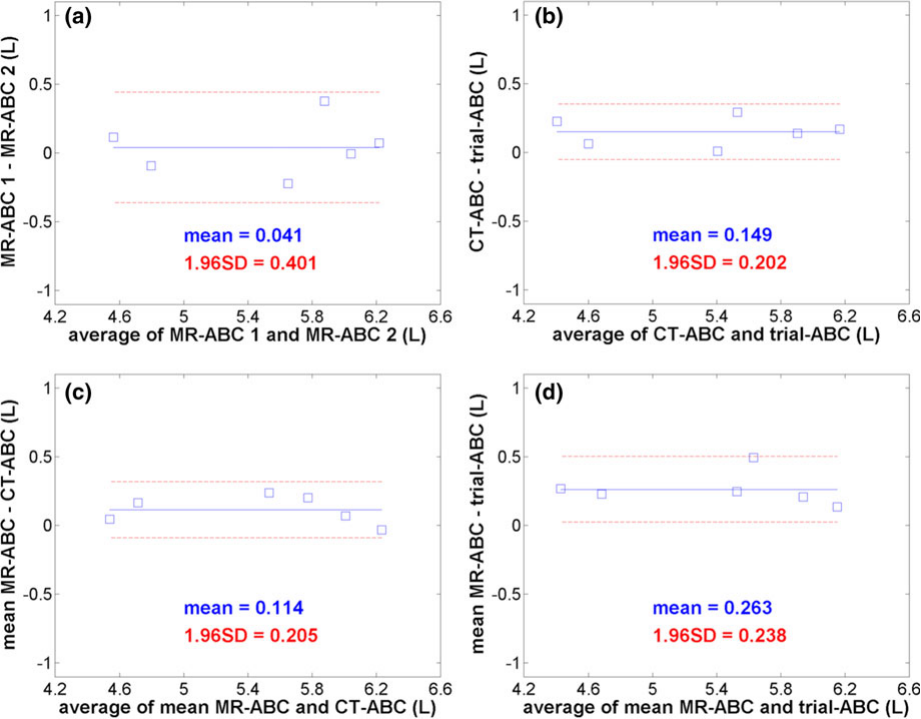
Bland‐Altman plots assessing the agreement of the mean of four lung volumes delineated from T_1_‐weighted 3D image datasets between ABC breath‐holding conditions over the six volunteers. (a) compares the two MR‐ABC sessions, (b) shows the volume comparison of the CT‐ABC to the trial‐ABC kit, while (c) and (d) compare the mean of the MR‐ABC sessions to the CT‐ABC and trial‐ABC, respectively. All plots have the same scale. The mean volume difference between two methods is numerically indicated in liters and as a blue solid line on every graph. The two dashed red lines denote the limits of agreement: mean volume difference ± 1.96 standard deviations (also mentioned on each graph in liters) of the differences for every comparison.

Bland‐Altman plots of the difference between the mean of four delineated lung volumes resulting from two ABC breath‐holding conditions against their average are displayed on Fig. 3. The mean difference between MR‐ABC‐controlled lung volumes on the two visits was 41 mL (0.7% of their average volume), and their agreement ranged within 400 mL (7.3% of their average volume around this value). The mean volume from both MR‐ABC sessions presented a greater bias against the CT‐ABC and the trial‐ABC volumes: 2.1% (114 mL) and 4.9% (263 mL), respectively, but a narrower band of agreement: 3.8% (205 mL) and 4.4% (238 mL) around the bias, respectively. The lung volumes arising from CT‐ABC or trial‐ABC control differed by 2.8% (149 mL) on average and agreed within 3.8% (202 mL) of their mean volume. In summary, the average of the limits of agreement over all ABC condition comparisons was 4.8% (262 mL) of the overall average volume.

## 4 Discussion

In this work, we took advantage of the recently available MR‐compatible ABC system to investigate the reproducibility of breath‐held lung volumes under ABC control while avoiding ionizing radiation exposure. The MR‐compatible apparatus offers new opportunities to address unanswered questions involving respiratory control and to repeat measurements with more image contrast options than CT without radiation concerns. The dependence of ABC‐displayed inhaled air volume on flow rate reported by McNair et al.^9^ may raise questions about lung volume reproducibility with ABC, which constitutes the device's respiratory control principle. Such concerns were addressed in this study, by employing a direct lung volume measure via accurate volumetric 3D imaging and delineation of the lungs, and an actual clinical setting with the same volunteer setup and imaging sequence as during MRI in lung cancer. The results of ABC‐induced compared to self‐sustained breath‐holding volume reproducibility can inform the choice of respiratory control method for clinical examinations. The evaluation of possible lung volume variations between similar clinically applied ABC kits with the same respiratory control settings served as their quality assurance regarding the clinical use of the device. Detailed component testing, calibrations and bench quality assurance of ABC systems are beyond the scope of this article.

Lung volumes were deduced from volumetric T_1_‐weighted MR image datasets using a semi‐automated ROI contouring method available within the widely used TPS RayStation. The large amount of acquired MR data (120 volumetric image datasets) was divided equally between three observers for lung delineation. For consistency, all 20‐image datasets of the same subject were processed by the same observer. Inter‐observer variability was estimated as a few percent of the calculated lung volume, using the four datasets of a volunteer and breath‐holding condition with typical image quality, and those of a case presenting imaging artifacts. Bland‐Altman analysis indicated that bias between observers may reach 4% while variation may be as much as 6%. The smaller bias between Observers 1 and 2 suggests that they followed a more similar lung contouring procedure than Observer 3. Image quality appeared to have a smaller impact on variability than the inevitable subjectivity of manual editing around ambiguous structures such as vessels and the diaphragm, which the automated algorithm cannot accurately contour.

This work presents the raw data of the calculated lung volumes from all subjects and breath‐holding conditions, and summary statistics. Performing four breath‐holds allowed the variability between breath‐holds to be assessed for each condition, and averaging these volumes improved the accuracy of comparisons between the different conditions. The total number of performed breath‐holds in one session should be restricted to avoid volunteer fatigue, and reached 16 in our study. The duration of the induced breath‐holds was close to the clinical limit of 20 s. Given the small number of subjects we implemented descriptive statistics only, avoiding hypothesis testing.

Our data suggest that the ABC‐controlled breath‐holding volumes were more closely clustered than the self‐sustained ones. The overall ABC intra‐session lung volume variability (1.8% of the mean lung volume attained using ABC), was less than half the self‐sustained intra‐session lung volume variability. However, one subject (Volunteer 5) presented the largest standard deviation for self‐controlled breath‐holding (9.3% of his mean lung volume), thus increasing the average standard deviation of self‐sustained volumes over the rest of the subjects (3.0%) to the reported 4.1% average standard deviation of self‐sustained volumes over all subjects. Registration of images with a very small difference of delineated lung volumes under ABC control indicated very good overlap of all structures and lung contours. In contrast, self‐controlled breath‐holds with a large difference between their delineated lung volumes presented a poorer image and lung contour match, with considerable variation in diaphragm, vessels and thorax position.

Bland‐Altman analysis, investigating combinations of ABC breath‐holding conditions, revealed that lung volumes measured with MR‐ABC during different sessions agreed within 7%, with a 0.7% bias. Comparisons between the mean MR‐ABC volume values from the two visits and those obtained from the other two ABC kits yielded a slightly larger bias, but narrower limits of agreement. This outcome might be explained by minor differences in technical characteristics of the tested ABC kits and by minor physiological changes to the volunteers’ lungs or to the scanner performance between the two MR‐ABC scanning dates. The overall 5% limits of agreement obtained with ABC are very close to the average inter‐observer agreement value of 4%, which implies that lung delineation uncertainties may have been the main cause of the overall divergence of the ABC results. The small sample size may have also contributed to the observed lung volume variability.

The commonly used self breath‐holding method allowed subjects to hold each breath at an individually preferable inspirational depth. It is therefore not directly comparable to the voluntary DIBH method applied in Bartlett et al^10^, where breath‐hold consistency was verified by patient tattoo positions, but is similar to the coached DIBH maneuvers in Hanley et al^15^, except that the inspirational level was not specified in our case. Our work suggests that application of the ABC apparatus ensures reproducible lung volume levels and shapes for persons less able to accurately control their own breath‐holds. Some individuals are capable of replicating their own breath‐holds well, but not necessarily reaching the same lung volumes as under ABC control. The advantage of the ABC apparatus is that it can enforce an individually specified lung volume level to all subjects.

The volunteers who participated in this study were healthy, compliant and breathed regularly. We presume that non‐ABC trained persons, especially lung cancer patients, would present a larger variation and more pronounced inconsistencies in lung volumes attained in the standard clinical practice of operator‐guided self‐controlled breath‐holding. Some individuals may have faster, slower or more irregular breathing patterns than operators assume, and may not be able to adhere to instructions. As scanner operators usually have no visual cues of the patient respiratory traces, patients may be breathing during parts of the scanning. This source of lung volume uncertainty was eliminated during our experiment, where operators were observing the volunteer respiratory traces displayed by the ABC control software and started MR acquisition at the actual breath‐hold onset. The automated external triggering option described in Kaza et al^6^ was explicitly not employed in this work, to imitate the operator‐induced CT scanning and irradiation using ABC in radiotherapy, and to maintain the same operator‐prompted MR acquisition method between self‐controlled and ABC‐controlled breath‐holding.

A limitation of our study is that the number of volunteers (6) and sessions (2) was small. Nevertheless, this work is valuable as an exploratory study, and the 24 acquired datasets for each of the five conditions are sufficient for its purposes. Furthermore, for organizational reasons we could not test the entire CT and breast trial‐ABC systems, including their control modules and connectors. However, the assessed components (digital volume transducer & pickup assemblies, balloon valves with their tubing) are the most sensitive parts, which predominantly contribute to volume measurements and breath‐hold onset and stability, and are hence expected to cause the largest amount of possible variations between different systems. In order to reach the ABC control module outside the RF panel, all original ABC balloon valve pneumatic tubes were elongated by 3 m, leading to about 0.7 s increase of balloon valve inflation delay.^6^

The results of our investigation are expected to be instructive for various respiratory control applications. Depending on the necessary degree of lung volume reproducibility, one can decide to employ the more accurate and elaborate ABC apparatus or the easier applicable self‐controlled breath‐holds, to meet different research or clinical examination requirements. If attaining a particular lung volume were crucial, as for radiotherapy treatment planning or for combining images produced from different modalities, the ABC would be the method of choice. Furthermore, our findings of good reproducibility over entire lung volumes under ABC control are very important for radiotherapy treatment planning, as they suggest a good position reproducibility of structures inside and around the lungs and consequent reduction of treatment margins. The lung volume agreement achieved with ABC between sessions and using devices applied for different imaging modalities indicates that images obtained on different dates from different modalities under ABC control are comparable. Moreover, as ABC represents the most established breath‐hold replication method, its lung volume reproducibility portrays the level of currently attainable lung volume reproduction accuracy by halting respiration. Images acquired in ABC‐controlled breath‐holds at specific respiratory phases may serve as reference for image‐guided applications such as the MR‐linac.

## Conclusion

The reproducibility of lung volumes attained during MRI in ABC‐controlled and operator‐guided self‐controlled breath‐holds was assessed for the first time in healthy volunteers. Segmented images of lung volumes differed by 3% on average and agreed within 7% between two sessions using the same ABC components, suggesting good inter‐session reproducibility. Three different clinically applied ABC kits, used for MRI, CT, and breast trial treatment, yielded a similar kit intra‐session lung volume variability. Overall ABC intra‐session lung volume variation was 1.8% (99 mL), about half of the 4.1% (226 mL) variability observed on average with self‐sustained breath‐holding.

## Conflict of interest

The authors have no conflicts of interest to disclose.
